# Alzheimer’s disease as a primary pathology in logopenic variant of primary progressive aphasia

**DOI:** 10.1002/alz.089446

**Published:** 2025-01-09

**Authors:** Kseniya Nevzorova, Yuliya Shpilyukova, Diliara Akhmadullina, Alla Shabalina, Ekaterina Fedotova

**Affiliations:** ^1^ Research Center of Neurology, Moscow Russian Federation

## Abstract

**Background:**

The logopenic variant of primary progressive aphasia (lvPPA) is a disorder centered on language impairment. Alzheimer Disease (AD) is one of the most common underlying pathologies in lvPPA. The aim of our study was to estimate frequency of AD in this phenotype and to analyze clinical features of AD‐lvPPA.

**Method:**

We examined 20 patients (9 females and 11 males, 65.9±9.1 years) with the phenotype of lvPPA. The diagnosis was determined on clinical and imaging data according to the lvPPA criteria (Gorno‐Tempini et al., 2011). All patients underwent lumbar puncture. CSF biomarkers Abeta‐42 and p‐tau181 were analyzed. Based on the laboratory results and according to the recommendations of International Working Group (2021) we found 4 patients with established AD, 8 patients with probable AD and 8 non‐AD patients. Then we compared 2 groups (AD and non‐AD) on age of onset and cognitive features, assessed by Addenbrooke’s cognitive examination (ACE‐R) and Frontal Assessment Battery (FAB).

**Result:**

The frequency of AD pathology in lvPPA group was 60% (20% for established diagnosis and 40% ‐ for probable). The disease onset was earlier in the group of AD lvPPA than in the non‐AD group (60.1 years vs. 65.4 years), but the difference was not statistically significant (p=0.175). We didn’t find significant differences between the groups in FAB, total ACE‐R. We revealed a higher median score in the speech ACE‐R subscale (18.5 vs. 14.5) and lower median score in the memory ACE‐R subscale (10.5 vs. 14.5) in patients with established AD group compared to non‐AD group.

**Conclusion:**

In our study we demonstrated a high prevalence of AD pathology in lvPPA. The results showed no specific differences, but some clinical findings may predict AD on pre‐laboratory stage (such as more severe memory impairment and less speech impairment). So, it is important to analyze AD biomarkers in CSF in all cases to establish underlying pathology and to prescribe specific treatment.

The study was supported by RSF (#23‐25‐00483)

